# Milk Bioactive Compounds and Gut Microbiota Modulation: The Role of Whey Proteins and Milk Oligosaccharides

**DOI:** 10.3390/foods13060907

**Published:** 2024-03-16

**Authors:** Valentina Gallo, Alyexandra Arienzo, Federica Tomassetti, Giovanni Antonini

**Affiliations:** 1 Department of Science, Roma Tre University, Viale Guglielmo Marconi 446, 00146 Rome, Italy; federica.tomassetti@uniroma3.it (F.T.); giovanni.antonini@uniroma3.it (G.A.); 2 National Institute of Biostructures and Biosystems (INBB), Viale delle Medaglie d’Oro 305, 00136 Rome, Italy; alyexandraarienzo@gmail.com

**Keywords:** gut microbiota, whey proteins, lactoferrin, lactalbumin, lysozyme, lactoperoxidase, glycomacropeptide, milk oligosaccharides, prebiotics, probiotics

## Abstract

A strong correlation between the occurrence of various pathological conditions and intestinal dysbiosis is supported by a range of strong evidence. Vice versa, many pathologies have been shown, in turn, to be responsible for alterations in the gut microbiota, a condition that can worsen illness outcomes and response to therapies. For these reasons, great efforts have been made, and studies are still ongoing, to elucidate the mechanisms underlying gut microbiota alterations and to search for pharmacologic or other strategies that can effectively restore the gut microbiota. In this narrative review, we examined the most significant literature on the role of some milk bioactive compounds, such as milk oligosaccharides and whey proteins, in modulating the composition of the gut microbiota and the underlying mechanisms of action, with the aim of investigating the impact of the microbiota changes mediated by these milk bioactive molecules on human health, and their potential use as therapeutics to treat or adjuvate the treatment of gut dysbiosis and associated pathologies.

## 1. Introduction

The term microbiota defines the entire microbial community (including commensal, mutualistic or pathogenic) that inhabit a given habitat; in this sense, gut microbiota refers to the range of microorganisms (i.e., Archea, Bacteria, Eukarya, and viruses) which reside in the gastrointestinal (GI) tract. Billions of symbiotic microorganisms populate the human gut, presiding over structural [1,2], protective [3,4,5], neurological, and metabolic functions [6,7,8], within such an intimate and intricate relationship that it gives rise to a unit called the holobiont [9]. The gut microbiota is acquired at birth and is characterized by great instability in terms of structure and composition throughout childhood [10]. After childhood, the gut microbiota, still maintaining very dynamic behaviour, begins to differentiate into a more defined structure [11,12,13]. Indeed, adult gut microbiota is characterized by a quasi-constant composition of those microbial species which are more favoured in the gut microenvironment [14,15]. For example, the most represented gut microbiota bacteria belong to Firmicutes, Bacteroidetes, Actinobacteria, Fusobacteria, Proteobacteria, Verrucomicrobia, and Cyanobacteria phyla, where Firmicutes and Bacteroidetes constitute more than 90% [16]. It is noteworthy that the Firmicutes/Bacteroidetes ratio plays a key role in influencing intestinal homeostasis. Indeed, variation in the Firmicutes/Bacteroides ratio is strongly associated with gut dysbiosis and associated diseases, including obesity and bowel inflammatory disorders [17]. However, the relative abundance of bacterial phyla and/or colonisation by other microorganisms may vary greatly depending on all the factors that can influence the gut microenvironment parameters (e.g., pH, oxygen levels, temperature, availability of nutrients). These factors include dietary habits [18], age [19], pharmacological therapies (e.g., antibiotics) [20,21,22], host genetics [23,24], host geographic location [25], pathologies, lifestyle, and environmental stress [26,27], and account for high intra-individual time-related variations and for inter-individual diversification [28,29,30]. Compared to the microbiota which colonise other body sites (e.g., oral mucosa, vagina, and skin), gut microbiota is the major focus of researchers’ interest due to its higher clinical significance [31]. Indeed, studies have shown that the equilibrium between health status and the arising of a disease state is strongly influenced by the composition of microbiota, particularly by the equilibrium between the different species and the predominance of some of them [32]. Despite the difficulties in defining a standard health promoting microbiota composition, due to the great variability related to host and environmental factors, several studies report a strong correlation between gut dysbiosis, which leads to a decrease in microbial diversity, and the occurrence of various pathological conditions [33,34,35]. These include inflammatory and metabolic disorders, obesity, and type II diabetes [7,36], but gut dysbiosis has also been associated with the promotion of some types of cancer, including colorectal cancer [37,38], as well as neurological and neuropsychiatric disorders, including Alzheimer’s disease [39] and major depression [40,41], through the impairment of the gut-brain axis homeostasis [42]. Conversely, pathologies are often associated with the development of gut dysbiosis, which exacerbates the consequences of disease [43].

Great efforts to define the factors affecting the highly dynamic composition of the human gut microbiota and to better understand its role in health and disease have been made and are ongoing [44]. Based on these, therapeutic interventions aimed at restoring the establishment of a health-promoting gut microbiota could be useful for the prevention and treatment of gut dysbiosis. Diverse strategies, ranging from the use of probiotics, prebiotics, and pharmaceutical compounds to non-pharmacological approaches, such as faecal microbiota transplantation, have been shown to be effective in positively modulating the microbiota [30,45,46,47,48]. Importantly, one area that has gained increasing interest in recent years is the role of prebiotics in these processes. Prebiotics are a non-digestible group of molecules derived from food sources that are selectively fermented by probiotics, health beneficial gut microorganisms (e.g., *Bifidobacterium* and *Lactobacillus* genera), stimulating their growth and inducing beneficial changes in the gut microbiota composition; importantly, their effects on maintaining an optimal Firmicutes/Bacteroides ratio are well documented [49].

In this context, milk and its bioactive components have long been studied for their potential as prebiotics. Indeed, due to its content of bioactive molecules [50], including proteins, lipids, and oligosaccharides, milk is considered as a functional food whose antimicrobial [51], immunological [52], and antitumorigenic activities have been widely studied and exploited in nutraceutical and biomedical fields to support the treatment and prevention of diseases [50,53,54,55,56,57,58].

Infant gut microbiota develops at birth and breast milk plays a key role in this process [59]. Several studies have linked changes in dietary habits during pregnancy to alterations in the maternal and in the infant gut microbiota [60,61], and a marked difference between gut microbiota of breast-fed and formula-fed infants has been demonstrated in metagenomic studies [11], which indicate that the breast-fed infants’ microbiota is less diversified but consists mainly of health-beneficial bacteria such as bifidobacteria, lactobacilli, and staphylococci [62,63,64]. Apart from the role exerted by its own microbiota, which contributes to a healthy microbial diversification of the gut microbiota of newborns [65,66], the bioactive components of milk, especially oligosaccharides and whey proteins such as lactoferrin, lysozyme, and α-lactalbumin, have been shown to play a crucial role in shaping the gut microbiota from birth to adulthood [67,68]. Indeed, studies have reported the role of human and cow’s milk constituents in promoting the growth of beneficial probiotic bacteria, including *B. infantis*, *B. Pennsylvanicus*, *B. longum*, *B. bifidum*, and *B. breve* [69,70,71,72].

In this work, we reviewed the most significant literature on the role of milk bioactive compounds in influencing and modulating the composition of gut microbiota in newborns and throughout life, focusing on oligosaccharides and whey proteins. We have also evaluated the impact of the microbiota changes mediated by these milk bioactive molecules on health. This article aims to provide and spread up-to-date knowledge on this promising and evolving field of research and to raise awareness of the main current limitations. This may be helpful to stimulate further research aimed at improving our understanding of the prebiotic effects of these molecules, of the underlying mechanisms of action and of their potential beneficial effects on health, and to open the possibility of using these molecules, alone or in synergy with other drugs, as therapeutics to treat and/or adjuvate the treatment of gut dysbiosis and related diseases.

## 2. Prebiotic Activity of Milk Oligosaccharides

### 2.1. Mechanisms of Action

Milk consists of three main structural classes of oligosaccharides: fucosylated, non-fucosylated neutral, and sialylated, which are composed of five sugar residues with a variable degree of polymerization. Human milk has a higher content of oligosaccharides than cow’s milk, which is about 20-fold lower; furthermore, fucosylated and neutral species are predominant in human milk, whereas sialylated species are most abundant in cow’s milk [73,74,75]. Two main mechanisms have been associated with the modulation of the gut microbiota by milk oligosaccharides (MOs) [76]. The first mechanism is related to a direct prebiotic activity of MOs, which have been shown to selectively promote the growth of certain *Bifidobacterium* strains, including *B. infantis, B. breve*, and *B. bifidum*, as well as some *Lactobacillus* species [77,78,79,80,81]. Furthermore, in vitro studies have shown that structural differences in MOs account for their selective utilization by gut microbiota bacteria [82,83]. The second mechanism is related to the inhibition of colonisation by various enteric pathogens, including *Vibrio cholerae*, *Salmonella fyris*, *Campylobactor jejuni, Clostridioides difficile*, and various *Escherichia coli* strains, by preventing epithelial adhesion (Table 1). Indeed, studies demonstrated the capability of MOs, especially those belonging to the fucosylated class, to compete with pathogens in binding to host cell receptors and to sequester pathogens by acting as free analogues of host cell receptors [84,85,86,87].

### 2.2. Effects of MOs in Modulating Gut Microbiota and Health: In Vitro Studies and Clinical Trials

In addition to a protective role against diverse enteric pathogens, studies suggest that the effects of MOs on gut microbiota can lead to a modulation of the immune system, reducing the risk of allergies, asthma, and inflammatory diseases [88]. Indeed, a deficiency in bifidobacteria, and thus in the genes required for the metabolism of MOs, has been shown to correlate with systemic inflammation and immune system dysregulation in infants [89,90,91]. Furthermore, MOs have been demonstrated to be important substrates for short-chain fatty acids production, particularly butyrate, which is mediated by an interplay between bifidobacteria and butyrate-producing bacteria [92,93]. This has deep health implications, since butyrate plays a critical role in maintaining the gut mucosal barrier and modulating the immune system of infants and adults. In addition, the bifidogenic effect of MOs is important for the production of other health-promoting metabolites, including the neurotransmitter GABA [94] and indole-3-lactic acid, which is involved in the activation of the aryl hydrocarbon receptor, a regulator of the gut-brain axis, intestinal homeostasis, and immune response [95]. However, in adults, the levels of bifidobacteria decrease greatly (up to 90%) and this has been demonstrated to be related to the insurgence of various diseases and GI disorders, including those associated with changes in intestinal permeability (leaky gut). In this context, in vitro studies and clinical trials to evaluate the effects of MOs in modulating adult gut microbiota have been made in an attempt to exploit these molecules as therapeutics for the restoration of a healthier gut [96,97,98]. In vitro studies by Šuligoj and colleagues investigated the effects of human MOs (HMOs) on the gut microbiota using Caco2 cell lines, human intestinal gut organoid-on-chips, and the Simulator of the Human Intestinal Microbial Ecosystem (SHIME^®^) as models. This study showed that the treatment with 2′-O-fucosyllactose (2′FL) and/or lacto-N-neotetraose (LNnT) fermented HMOs led to a significant upregulation of cytokines, permeability, and tight junction proteins, including claudin-8 and claudin-5, suggesting a role of HMOs fermented by gut microbiota in positively promoting gut barrier functionality [99]. Elison et al. performed a clinical trial based on oral supplementation of HMOs in 100 healthy, adult volunteers, providing sets of data on safety, tolerance, and impact of HMOs on the adult gut microbiota. In this study, chemically produced 2′FL and/or LNnT at various daily doses and mixes, or placebo, were administered for 2 weeks. The obtained results showed that the uptake of 2′FL and LNnT is sufficient to modulate the adult microbiota within two weeks, with an increase in relative abundance of bifidobacteria, to >25% in some individuals, and a reduction in the relative abundance of Firmicutes and Proteobacteria phyla [100]. In another study, the effects of oral supplementation of 2′-O-fucosyllactose and lacto-N-neotetraose HMOs on gut microbiota were evaluated in patients with irritable bowel syndrome; a modulation of the gut microbiota, and of faecal and plasma metabolite profiles, associated with the HMOs bifidogenic effect, was observed [101].

All these studies highlight the beneficial effects of HMOs beyond childhood, suggesting their potential in supporting the restoration of a wholesome adult gut microbiota. In this context, interesting results came from a more recent study which investigated the age-dependent impact of HMOs on gut microbiota. In this study, 6-year-old children and adults were enrolled for ex vivo testing using systemic intestinal fermentation (SIFR^®^) technology. Single types of HMOs, including 2′FL, LNnT, 3′Sialyllactose, 6′Sialyllactose, and combinations of these were used for this study. The results showed that age-dependent differences in microbiota composition, especially in terms of *Bifidobacterium* strains, strongly impact the utilization of HMOs and the individual response to the treatment, suggesting that the development of age-specific nutritional supplements using HMOs could maximize their beneficial outcomes [102].

**Table 1:** Prebiotic activity of milk oligosaccharides (MOs), mechanisms of action and potential health implications.

Activity	Mechanism of Action	MOs	Effects on Gut Microbiota	Potential Health Implications	Ref.
Direct prebiotic activity	Selective utilization by the bacteria of the gut microbiota of structurally different classes of MOs.	HMOs (2′-O-fucosyllactose; Lacto-N-neotetraose); sialic acid.	Promotes the growth of probiotics, including *Bifidobacterium* (i.e., *B. infantis*, *B. breve*, *B. bifidum*) and some *Lactobacillus* species.	Modulation of the immune system, reducing the risk of allergies, asthma, and systemic inflammatory conditions.	[78,79,80,82,83,89,90,91]
	MOs provide substrates which support the metabolism of diverse probiotics, especially *Bifidobacterium*, thus stimulating the production of health beneficial metabolites.		Promote the production of the neurotransmitter GABA and indole-3-lactic acid, a regulator of gut-brain axis by *Bifidobacterium (B. adolescentis*).	Modulation of the gut-brain axis response, intestinal homeostasis and immune response.	[69,94]
			Promote the production of short-chain fatty acids, especially butyrate, mediated by an interplay between bifidobacteria and butyrate-producing bacteria.	Maintenance of the gut mucosal barrier by limiting intestinal permeability (leaky gut); modulation of the immune system.	[92,93,99]
Indirect prebiotic activity	MOs, especially fucosylated classes, compete with pathogens in binding to host cells by acting as free analogues of cell receptors.		Prevent epithelial adhesion thus inhibiting colonisation by diverse enteric pathogens (i.e., *V. cholerae*, *S. fyris*, *C. jejuni*, *C. difficile* and *E. coli*).	Protective role against enteric diseases.	[84,85,86,87]

## 3. Prebiotic Activity of Whey Proteins

Milk contains two main classes of proteins: caseins and whey proteins. Human milk’s whey proteins include α-lactalbumin, immunoglobulins, serum albumin, lactoferrin, glycomacropeptide, lactoperoxidase, and lysozyme [103]. Bovine milk differs from human milk in the presence of β-lactoglobulin and lower levels of α-lactalbumin [104].

Whey proteins exert several functions including antioxidant, anticancer, antimicrobial, anti-inflammatory, and immunomodulatory [105,106,107,108,109,110,111]. Furthermore, evidence suggests a beneficial role of whey proteins in positively modulating the gut microbiota in both infants and adults [112,113,114].

### 3.1. Lactoferrin

Lactoferrin is an 80 kDa positively charged iron-binding glycoprotein, that belongs to the transferrin superfamily and is present at high levels in various biological fluids, especially in milk and colostrum [115,116]. Depending on the degree of iron saturation, it exists in two forms: the iron-depleted (apo) and the iron-saturated (holo). Both the apo and holo forms of lactoferrin and its peptide derivatives, including lactoferricin, play an important role in promoting and maintaining a functional gut microbiota and in inhibiting gut barrier impairment [117,118,119,120]. Experimental evidence suggests that these activities are mainly due to its strong antimicrobial activity, including bacteriostatic (related to the iron sequestering function) and bactericidal (linked to the binding and the consequent neutralization of some anionic bacterial surface components, such as lipopolysaccharide), as well as immunomodulatory functions [121,122,123], which help to preserve the integrity of the gut barrier [124,125].

#### 3.1.1. In Vitro Studies

**Figure 1:**
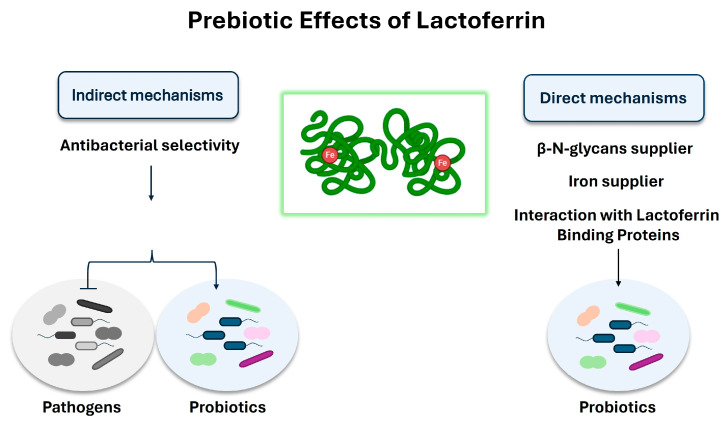
Prebiotic activity of lactoferrin: mechanisms of action. Both direct and indirect prebiotic effects have been described. The indirect mechanism is due to the lactoferrin-mediated iron sequestering that leads to a selective inhibition of the growth of pathogens, promoting the growth of those probiotics, such as *Bifidobacterium* and *Lactobacillus*, which do not necessarily need iron to grow. In the direct mechanism, lactoferrin promotes the growth of probiotics (especially *Bifidobacterium*) by acting as β-N-glycans and iron supplier, or by interacting with the lactoferrin-binding proteins present on the membrane of some probiotics, including *Bifidobacterium* and *Lactobacillus*. This interaction has been shown to promote the cytosolic internalization of lactoferrin which has been suggested to modulate genes involved in DNA replication and cell growth.

Various in vitro studies suggest a prebiotic role of lactoferrin and its peptide derivative, lactoferricin, on the gut microbiota [126,127] (Figure 1). Though the mechanisms still need to be better clarified, indirect prebiotic effects have been observed, mainly related to a selective inhibition of bacterial growth. Indeed, some studies have shown that instead of directly promoting the growth of probiotics (i.e., Bifidobacteria and Lactobacilli) [128], lactoferrin acts mainly by inhibiting the growth of pathogens, which are less resistant to its antibacterial activity [129]. Lactoferrin-mediated iron sequestering could be one explanation for this antibacterial selectivity; indeed, contrary to many pathogens, such as *E. coli*, various probiotics, including many Bifidobacterial strains, do not necessarily need iron to grow and are able to predominate over pathogens under conditions of iron deprivation [130,131,132]. However, various studies have also shown direct prebiotic effects; in this context, different mechanisms of action have been postulated from the results of in vitro studies. Some studies suggest a role for lactoferrin as a supplier of β-N-glycans to stimulate bifidobacterial growth [133], while other in vitro studies have shown that the holo form of lactoferrin promotes the growth of *Bifidobacterium breve* by acting as an iron supplier [134]. In addition, some studies reported the presence of lactoferrin-binding proteins on the surface of some probiotics which are sensitive to the prebiotic action of lactoferrin, suggesting that the interaction between the cationic surface of lactoferrin and some anionic cell surface components may be involved in mediating this process [135,136]. Indeed, studies have shown that these interactions favour the cytosolic internalization of lactoferrin which, in the presence of ATP, dissociates to the N-lobe and C-lobe [137]; it has been suggested that the internalization of the C-lobe in the nucleus mediates the modulation of genes involved in DNA replication and cell growth [127].

However, regardless of the mechanisms involved, the results obtained from testing different experimental conditions indicated that the direct prebiotic activity of lactoferrin may vary depending on factors such as bacterial strain, temperature, oxygen levels, lactoferrin iron-saturation levels, and dosage [138,139,140,141,142]. Chen et al., while exploring in vitro the prebiotic potential of bovine lactoferrin in 14 probiotics, including *B. breve*, *L. coryniformis*, *L. delbrueckii*, *L. acidophilus*, *B. angulatum*, *B. catenulatum*, and *L. paraplantarum*, observed a negligible effect on probiotic growth at 37 °C, but a strong lactoferrin-mediated dose-dependent restart of growth at lower temperatures (i.e., 22 °C), suggesting that the direct prebiotic effects of lactoferrin become significant when the probiotic growth is retarded by cold stress conditions [143]. These findings could be of great significance, especially in all those environmental conditions or clinical features which cause probiotic growth inhibition or retardation; however, further studies, including in vivo studies and clinical trials, are needed to translate these results into a clinically relevant context. More recently, the same research group investigated the transcriptome profiles of *Lactobacillus rhamnosus* GG, treated with 1 mg/mL bovine lactoferrin at 22 °C, to elucidate the molecular mechanism involved in the prebiotic effect of lactoferrin on *L. rhamnosus* GG. The results showed that lactoferrin supplementation is associated with the transcriptional modulation of several genes that are involved in many central metabolic pathways. Increased transcription levels have been observed for genes encoding transporter proteins, including ABC transporter permease and ABC transported related proteins; genes involved in amino acid synthesis, DNA replication, and peptidoglycan biosynthesis; and genes encoding for proteins involved in carbohydrate metabolism and stress responses, including CspA, LytR, and XRE. On the contrary, decreased transcription levels have been found for some genes involved in purine and pyrimidine metabolism, biosynthesis of antibiotics, and secondary metabolites. However, since the prebiotic activity of lactoferrin is strongly strain-dependent, further studies on other probiotic strains could be useful to better understand the underlying mechanisms [144].

#### 3.1.2. In Vivo Studies and Clinical Trials

Several in vivo studies and clinical trials have investigated the effects of lactoferrin supplementation on the gut microbiota and its potential health-promoting effects (Table 2).

Many in vivo studies have been conducted using mice as animal models, documenting the role of lactoferrin supplementation in the positive modulation of the gut microbiota [120]. In experiments on vitamin D deficient mice, Wang and colleagues showed that oral administration of 100 to 1000 mg/kg body weight (BW) of bovine lactoferrin induced a significant change in the gut microbial community, favouring the growth of lactobacillus through a direct mechanism [145]. In another study, performed in high-fat diet-induced obese mice, the oral administration of 100 mg/kg BW of bovine lactoferrin for 12 weeks was shown to induce a significant increase of Bifidobacterium spp. in the faeces and a decrease of Enterobacterales, compared to controls. Furthermore, this treatment was shown to reduce inflammation and regulate lipid and glucose metabolism, highlighting the potential of bovine lactoferrin to improve microbiota dysbiosis and associated health conditions induced by a high-fat diet [146]. The in vivo prebiotic activity of lactoferrin has been demonstrated in other similar studies, which suggest a correlation between the lactoferrin-mediated improvement of dysbiosis and the amelioration of diverse pathological conditions, including obesity, inflammation, and metabolic disorders [125,147,148]. Studies also showed the potential of native and iron-saturated bovine lactoferrin in restoring the normal levels of some anti-inflammatory bacteria (i.e., *Bacteroidaceae*, *Prevotellaceae* and *Rikenellaceae*) in a mouse model of antibiotic-induced dysbiosis [149]. In addition, a recent study demonstrated a role of lactoferrin in alleviating cognitive impairment in western-diet induced obese mice by improving the gut-microbiota-brain axis, mainly inducing an increase in Bacteroidetes (i.e., *Roseburia*), which led to an inhibition of microglial activation and neuroinflammation [150].

Several studies have also been performed on piglets, as they have a greater biological similarity to humans compared to rodents [120,127]. In a study conducted on weaning piglets, the supplementation of bovine lactoferrin increased the amount of Lactobacillus and Bifidobacterium and decreased the abundance of *Escherichia coli* in the cecum, leading to an associated improvement of intestinal immunity and the gut barrier [151]. In another study, piglets administered lactoferrin orally (0.5 g per kg body weight per day) showed increased levels of *Lactobacillus* and a lower abundance of *Veillonella* and *Escherichia-Shigella* in the jejunum, and of *Actinobacillus* in the ileum [152]. Other similar studies confirmed the role of bovine and human native full-length lactoferrin in promoting a health beneficial modulation of the gut microbiota [153,154]. In one of these studies, healthy full-term piglets were employed as newborn models and administered with a combination of lactoferrin and probiotics to evaluate the impact of this treatment on the development of gut microbiota. Interestingly, results showed a decrease in the abundance of *Enterobacteriaceae* (frequently involved in neonatal infections and sepsis), and an increase in the levels of *Erysipelotrichaceae* and *Veillonellaceae* taxa and of butyrate-producing bacteria (i.e., *Faecalibacterium prausnitzii*), compared to controls [155]. In addition to studies that used native full-length lactoferrin, interesting results came from a study in which a recombinant fusion peptide constituted by the two main lactoferrin peptide derivatives, namely lactoferricin and lactoferrampin, was used as a dietary supplement; results showed that weaned piglets administered with 0.1 g/kg of recombinant fusion peptide increased the amount of Lactobacillus and Bifidobacterium in the chyme of the stomach, duodenum, jejunum, ileum, colon, and caecum [156]. Importantly, in all these studies, a reduction in the diarrhoea rate and an enhancement in the piglets’ trends of growth, together with an increased intestinal cell proliferation and maturation, were observed, which were associated with the lactoferrin-mediated balancing of gut microbiota [157,158].

Several clinical trials have explored the potential of lactoferrin supplementation in both infant and adult gut microbiota and its beneficial consequences on health [159]. In a double-blind, placebo-controlled, randomized trial, conducted in VLBW neonates in Italy, bovine lactoferrin supplementation, alone or in combination with the probiotic Lactobacillus rhamnosus GG, was shown to reduce the incidence of a first episode of late-onset sepsis, a severe complication which frequently affects premature neonates, by affecting the fungal progression from colonisation to infection [160]. Similar results were obtained in another randomized controlled clinical trial performed in Peruvian neonates [161]. Interestingly, another clinical trial conducted in preterm infants showed the efficacy of a recombinant human lactoferrin (talactoferrin, TLf) in reducing infection [162]. Further evidence from randomized controlled trials on enteral lactoferrin supplementation in preterm neonates has been recently reviewed by Pammi et al. [163].

The prebiotic and gut microbiota modulating effects of lactoferrin have also been evaluated in both healthy individuals and those suffering from various pathologies or undergoing therapies. Indeed, pathogenic gut dysbiosis has been observed during diverse therapies, including anticancer chemotherapy [164], and various diseases, including cancer and HIV infection, and it has often been associated with disease progression [165,166]. Results from some clinical trials suggested that lactoferrin may provide an adjunctive therapy to accelerate the process of gut microbiota restoration, thereby improving health outcomes and the response to therapy.

In a double-blind, placebo-controlled clinical trial, the impact of oral supplementation of lactoferrin on gut microbiota was evaluated in paediatric oncological patients undergoing chemotherapy. Results showed that lactoferrin, besides being well tolerated, played a role in favouring the promotion of gut microbiota homeostasis and in exerting a protective role against Enterococcus and other pathobiont colonisations [167].

In a randomized, double-blind, crossover clinical trial design, the treatment effects of oral recombinant human lactoferrin (1500 mg twice daily) in immunodeficiency virus–infected participants undergoing antiretroviral therapy and enrolled in an intestinal microbiome study, were investigated. In this study, insignificant changes to the intestinal microbiota were observed, although there was remarkable stability in the microbial community over time [168].

Results from a double-blind, placebo-controlled study conducted in healthy elderly women who were orally supplemented with bovine lactoferrin showed a significant increase in the relative abundance of Holdemanella in the faecal microbiota. Furthermore, an increase in the relative abundance of Bifidobacterium was observed when lactoferrin was administered in combination with active galactooligosaccharides [169].

**Table 2:** Lactoferrin’s effects on gut microbiota: in vivo studies and clinical trials.

Patients or Animal Models	Treatment	Effects on Gut Microbiota	Health Effects	Type of Study	Ref.
Vitamin D deficient mice.	Oral administration of bovine lactoferrin (100 and 1000 mg/kg BW).	Reduces the abundance of *Oscillibater*; increases the proportion of *Lachnospiraceae*, *Faecalibaculum*, and *Lactobacillus*.	Stimulates the expression of vitamin-D receptor by regulating gut microbiota; reduces serum levels of pro-inflammatory cytokines; enhances intestinal barrier function.	In vivo	[145]
Mouse models of colitis induced by dextran sulphate sodium salt.	Oral administration of bovine lactoferrin (100 mg/kg).	Phylum level: decreases Bacteroidetes and Firmicutes; increases Verrucomicrobia. Family level: decreases *Muribaculaceae* and *Lachnospiraceae*; increases *Akkermansiaceae*.	Alleviates colitis by improving the inflammatory response and the structure of the colon barrier in the colon.	In vivo	[125]
High-fat diet induced obese C57BL/6J mice.	Oral administration of bovine lactoferrin (100 mg/kg BW).	Restores the abundance of *Bifidobacterium* spp.	Reduces inflammation and regulates lipid and glucose metabolism.		[146]
Hight-fat diet induced obese C57BL/6J mice.	Oral supplementation of bovine lactoferrin (100 mg/kg BW) for 12 weeks.	Increases faecal *Bifidobacterium* spp.; decreases *Enterobacteriales* and *Bacteroidetes*.	Improvement of high-fat diet induced microbiota dysbiosis, improvement of hypercholesterolaemia and hyperglycaemia.	In vivo	[147,148]
C7BL-6 mice with clindamycin-induced dysbiosis.	Native bovine lactoferrin; iron-saturated bovine lactoferrin.	Promotes the growth of *Bacterioidaceae*, *Prevoellaceae* and *Rikenellaceae*.	Reverses clinamycin-induced dysbiosis.	In vivo	[149]
C57BL/6J mice models of western diet-induced cognitive impairment.	Oral supplementation of lactoferrin (50 mg/kg BW) for 16 weeks.	Increases Bacteroidetes (i.e., *Roseburia*).	Alleviates cognitive impairment by improving gut-microbiota-brain axis.	In vivo	[150]
Weaning piglets.	Oral supplementation of bovine lactoferrin (1 to 3 g/kg).	Increases *Lactobacillus* and *Bifidobacterium*; decreases the abundance of *Escherichia coli* in the cecum.	Enhances the growth performance; reduces diarrhoea rate by improving gut barrier and balancing intestinal microbiota.	In vivo	[151]
Suckling piglets.	Oral administration of lactoferrin (0.5 g/kg BW) daily for a week.	Increases *Lactobacillus* and decreases *Veillonella* and *Escherichia*-*Shigella* in the jejunum and *Actinobacillus* in the ileum.	Reduction of diarrhoea incidence and enhancement in the trends of growth by promoting the development of intestinal function and modulating the microbiota in the small intestine.	In vivo	[152,158]
Healthy full-term piglets.	0.5 g of probiotic (FloraBABY) and 100 mg of bovine lactoferrin.	Reduces the abundance of taxa commonly associated with sepsis in pre-term human infants (*Enterobacteriaceae*); increases the levels of *Erysipelotrichaceae* and *Veillonellaceae* taxa; increases butyrate producers (i.e., *Faecalibacterium prausnitzii*).	Improvement of gut microbiota and gut barrier.	In vivo	[155]
Weaned piglets.	Diet supplementation with 0.1 g/kg recombinant bovine lactoferrampin-lactoferricin fusion peptide.	Increases the number of *Lactobacillus* and *Bifidobacterium* in the chyme of the stomach, duodenum, jejunum, ileum, colon and caecum.	Improvement of intestinal microflora.	in vivo	[156]
Very low birth weight neonates.	Oral administration of bovine lactoferrin (100 mg day) alone or in combination with the Lactobacillus rhamnosus GG (6 × 10^9^ colony-forming units/day).	Affects the progression from bacterial and fungal colonisation to infection.	Reduction of the incidence of a first episode of late-onset sepsis.	Clinical trial	[160]
Preterm infants.	Enteral administration of recombinant human lactoferrin (talactoferrin, TLf) 150 mg kg/12 h from day 1 until day 28 of life.	Alteration of faecal microbiome with a reduction of gram-positive pathogenic bacteria.	Reduction in the rate of urinary tract infections, possibly associated with enteric prophylaxis with TLf.	Clinical trial	[162]
Paediatric patients undergoing chemotherapy.	Oral supplementation of bovine lactoferrin (200 mg/day) for two months.	Promotes gut microbiota eubiosis by containing the growth of pathobionts (e.g., *Enterococcus*) and modulating the abundance of other taxa relevant to intestinal health (i.e., *Akkermansia*).	Counteracts the onset of dysbiosis, thus ameliorating health and the response to therapy.	Clinical trial	[167]
HIV–infected patients undergoing antiretroviral therapy.	Oral supplementation of recombinant human lactoferrin (1500 mg twice a day).	No significant changes in gut microbiota composition.	Promotes a remarkable stability in the gut microbial community.	Clinical trial	[168]
Healthy elderly women.	Oral supplementation of bovine lactoferrin (1 g/day), alone or in combination with galactooligosaccharides and vitamin D.	Increases *Holdemanella* in the faecal microbiota; increases *Bifidobacterium* in combination with active galactooligosaccharides.	Not assessed^.^	Clinical trial	[169]

### 3.2. α-Lactalbumin

α-Lactalbumin is a 14 kDa globular protein produced by the mammary glands’ epithelial cells and involved in the regulation of lactose synthesis [170]. α-Lactalbumin is the most prevalent whey protein in human milk, constituting approximately 35%, while its quantity is lower in bovine milk, representing the second most abundant whey protein (approximately 17%) after β-lactoglobulin (which is absent in human milk). Due to its nutritional and therapeutic properties, α-lactalbumin is used as a component of infant formula and as a supplement to modulate gastrointestinal and neurological functions and ameliorate diverse disease conditions [171,172]. Even though the mechanism of action underlying these health-promoting effects of α-lactalbumin remains to be elucidated, several pieces of evidence suggest that a pivotal role is played by α-lactalbumin bioactive peptides [173]. Importantly, α-lactalbumin and some of its bioactive peptides have been shown to play a role in the modulation of the gut microbiota by acting as prebiotics (Table 3), and studies suggest this is related both to a direct promotion of probiotics’ growth, including bifidobacteria, and, indirectly, to their antimicrobial activity against diverse pathogens [172,174,175,176]. Xie et al. demonstrated that hyperuricemic mice orally administered with α-lactalbumin gastrointestinal hydrolysates showed a reduction in the levels of serum uric acid, creatinine, and urea nitrogen in association with an increased abundance of some SCFA-producing bacteria, and a decrease of the growth of hyperuricaemia- and inflammation-associated genera [177]. In a previous study, Xie and colleagues demonstrated that treatment with α-lactalbumin hydrolysates is effective in alleviating hypertension-associated intestinal microbiota dysbiosis in spontaneously hypertensive rats [178]. In a study by Chen and colleagues, the effects of treatment with the α-lactalbumin peptide Asp-Gln-Trp in high-fat diet (HFD)-induced NAFLD mice were investigated; results showed that the treatment positively modulated the gut microbiota, reducing the relative abundance of pathogenic bacteria and enhancing the relative abundance of Firmicutes and short-chain fatty acid (SCFA)-producing bacteria [179]. Li et al. demonstrated that bovine α-lactalbumin hydrolysate alters gut microbiota by modulating the Bacteroidetes/Firmicutes ratios and increasing the relative abundance of *Lachnospiraceae* and *Blautia* in HFD-induced obese mice. Additionally, this supplementation was demonstrated to reduce the levels of inflammatory cytokines, such as interleukin-6 and tumour necrosis factor-α, and of lipopolysaccharides, leading to a significant reduction of obesity-associated systematic inflammation and endotoxaemia [180]. In another study, performed on high-fat diet-fed mice, the effects of dietary α-lactalbumin on intestinal–adipose–hypothalamic control of energy balance were investigated. Results showed that the supplementation led to a decrease in cd36 and glut2 gene expression in the intestine accompanied by increased cumulative energy ingestion. This was associated with a significant modulation of the gut microbiota with a higher abundance of the *Lactobacillus*, *Parabacteroides* and *Bifidobacterium* genera, compared to controls [181]. In another study, the effects of α-lactalbumin supplementation were tested in preterm pigs that were used as a model for newborn infants. In this study, pigs receiving diets with a high content of α-lactalbumin had a higher abundance of Clostridiaceae, Enterobacteriaceae, *Streptococcus*, and *Streptomyces*, compared to controls, but no differences were found at the class or phylum level [182].

Other studies have demonstrated that the health-promoting properties of α-lactalbumin, associated with its effects on positively modulating gut microbiota, can be enhanced when the protein is used in combination with other drugs and/or other milk bioactive components. In a randomized controlled trial, the addition of 3.0 g/L of oligofructose to an α-lactalbumin-enriched term infant formula led to a synergic effect, resulting in a greater increase in faecal bifidobacteria compared to infants receiving the control formula without oligofructose [183]. In a pilot, open label, controlled and interventional study, the authors showed that the oral supplementation with a combination of inositols, α-lactalbumin, and *Gymnema sylvestre*, positively impacts insulin, glucose, lipid metabolism, and anthropometric measures in dysmetabolic and obese patients, and suggested a role of α-lactalbumin in recovering gut dysbiosis and related metabolic effects [184].

### 3.3. Lysozyme

Lysozyme is a 14.4 kDa antibacterial enzyme found in plants and animals [185]. In animals, lysozyme is present in diverse biological fluids, including saliva, tears, and milk [186]. Human milk, especially colostrum, has a high content of lysozyme (ranging from 0.2 to 0.9 g/L, depending on lactation stage), while its content is significantly lower in bovine colostrum, with only traces detectable in mature bovine milk [187,188].

As for lactoferrin, the antibacterial activity of lysozyme has a role in reducing the complexity of the gut microbiota, increasing the resistance to intestinal colonisation by some bacterial species, including pathogens, still favouring the growth of beneficial bacteria, and enhancing the recovery from diverse gastrointestinal pathological conditions [189,190]. The mechanisms underlying the resistance of some probiotic strains to the antibacterial action of lysozyme is not yet completely elucidated. However, results from in vitro studies on human-residential bifidobacteria indicated that the tolerance among some bifidobacteria strains is to be ascribed to the non-enzymatic antibacterial activity of lysozyme [191].

Several in vitro and in vivo studies have shown that lysozyme is efficient at modifying the gut microbiota [192,193,194,195] (Table 4). Human lysozyme was found to significantly reduce the number of coliforms and *E. coli* in the intestine of young pigs fed with milk from transgenic goats expressing human-comparable levels of human lysozyme, compared with pigs fed with milk from non-transgenic goats [196]. Results from similar studies showed that the presence of human lysozyme in goat milk ameliorates intestinal inflammation [197], results in the inhibition of pathogenic *E. coli* growth in young pig models, and accelerates recovery from *E. coli*-induced diarrhoea [198]. In addition to modulating pigs’ gut microbiota by decreasing disease-causing bacteria numbers, lysozyme transgenic goat milk was found to increase the ratio of beneficial bacteria, and these changes were associated with improved gut health. The analyses of faecal microbiota showed changes in the bacterial abundance at levels of Firmicutes and Bacteroidetes phyla, with a decrease of Firmicutes and an increase in Bacteroidetes, as well as a reduction of clostridia, *Streptococcaceae*, and disease-related bacteria such as *Mycobacteriaceae* and *Campylobacterales*, in pigs fed with lysozyme transgenic goat milk compared to controls. Furthermore, the presence of lysozyme in goat milk resulted in a gut microbiota more similar to that of breast-fed human infants, which presented a major abundance of *Bifidobacteriaceae* and *Lactobacillaceae* [199]. To support these results, other studies have evaluated the effects of lysozyme deficiency on the occurrence of intestinal dysbiosis. The production of lysozyme by Paneth cells is known to be a key factor in gut microbiota modulation and stabilisation [200]. Indeed, deficiencies in the secretion of lysozyme by Paneth cells have been shown to be related to gut microbiota disorders and to an increased vulnerability to bacterial infections and intestinal inflammation [201,202,203]. Interestingly, results from an in vivo study performed on mice with a deoxynivalenol-induced depletion of Paneth cells, demonstrated that supplementation with lysozyme was effective in improving Paneth cells’ functionality and gut microbiota restoration [204].

## 4. Prebiotic Activity of Glycomacropeptide

Glycomacropeptide (GMP) is a 64-amino acid glycosylated bioactive peptide derived from the C-terminal region of kappa-casein [205]. GMP is mostly found in dairy products, being released in whey by enzymatic digestion during cheese-making processes [206]. However, it is also found free in cow milk and whey, though in lower amounts [172,207].

GMP’s extensive glycosylation is responsible for most of its biological properties [208], which include antibacterial, anti-tumoral, and immunomodulatory effects [205,209]. Interestingly, studies showed that glycosylation, particularly oligosaccharide chains with a high content of sialic acid, accounts for the GMP prebiotic activity, which has been suggested to be responsible for GMP’s diverse biological activities and beneficial health effects [210]. Aside from numerous in vitro studies that demonstrated the efficacy of GMP in inducing the growth of probiotics such as *B. infantis*, *B. breve*, and *B. bifidum* [211,212], in vivo studies and clinical trials have also investigated the prebiotic effects of GMP (Table 5), and the first in vivo demonstrations came from studies performed in mice orally administered GMP. In a study, the treatment induced a significant decrease in faecal Enterobacteriaceae and coliforms together with an increase in *Lactobacillus* and *Bifidobacterium* [213]. In another study, it was observed that GMP induces modulation in the gut microbiota, mainly by reducing the growth of *Desulfvibrio* and increasing Firmicutes, and leads to an increase in caecal concentrations of SCFAs [210]. Experiments performed in allergen (ovalbumin)-sensitized rats orally treated with GMP demonstrated that GMP administration exerts a prebiotic action on allergy-protective microbiota bacteria, increasing the amount of *Lactobacillus* and *Bifidobacterium* after 3 days of treatment, and of Bacteroides after 17 days of treatment, suggesting that this, together with other GMP-induced responses, could have a role in the antiallergic activities of GMP [214]. Moreover, GMP hydrolysate was demonstrated to increase the Bacteroidetes/Firmicutes ratio and the abundance of S24-7, *Ruminiclostridium*, *Blautia*, and *Allobaculum*, in high-fat diet-fed and streptozotocin-induced type 2 diabetes mouse models, and these gut microbiota changes were associated with the observed antidiabetic effects [215].

Referring to the GMP effects in modulating human microbiota, a study performed using an artificial colon model of elderly gut microbiota showed that GMP sustained microbiota diversity, decreasing the abundance of *Clostridium* cluster IV and *Ruminococcus* and favouring the growth of *Blautia spp*. [216], suggesting that GMP could exert even in humans the strong prebiotic activity that was observed in in vitro and animal studies. This hypothesis was confirmed in some clinical trials. In a prospective, non-randomized, controlled trial, alterations of gut microbiota, consisting of a significant increase in the abundance of *Bifidobacterium*, were observed in very preterm infants fed with an infant formula containing a specific prebiotic mixture of 0.65 g scGOS/lcFOS (9:1) and casein GMP providing 40 mg sialic acid/100 mL; these microbiota changes resulted in a more efficient production of neuroactive compounds and energy source utilization [217]. Furthermore, in a two-week clinical trial performed on obese postmenopausal women, alteration in the faecal microbiota, consisting of a reduction of members of the genus Streptococcus and of overall α diversity, was observed after a supplementation with 15 g GMP + 10 g whey protein twice daily for one week, and three times daily for one week, respectively. This study showed that the supplementation of GMP, in combination with other whey proteins, improves satiety and regulates glucose homeostasis, suggesting its potential role as a helpful nutritional supplement for the reduction of metabolic syndrome risk in obese postmenopausal women [218]. However, in other clinical trials, no significant alterations in the gut microbiota were observed after GMP treatment [219,220]. One of these studies was performed in adult subjects with irritable bowel syndrome, and no significant changes in faecal microbiota and faecal immune markers were observed after a three-week period of daily supplementation with 30 g of GMP. The authors suggested that diverse factors, including species-specific gene expression variations, may account for the divergent results obtained from in vitro and animal studies, compared to those from clinical trials [221]. However, all the studies discussed above suffer from some limitations, including interindividual variability and sample size, and further clinical trials are needed to fully assess the role of GMP in modulating the human gut microbiota.

**Table 5:** In vivo studies and clinical trials on the modulation of gut microbiota by glycomacropeptide (GMP).

Patients or Animal Models	Treatment	Effects on Gut Microbiota	Health Effects	Type of Study	Ref.
Male BALB/c mice.	Oral administration of GMP (0.5 mg/mL at a dose of 0.2 mL per day).	Decrease in *Enterobacteriaceae* and coliforms; increase in *Lactobacillus* and *Bifidobacterium*.	Establishment of a healthier intestinal microbiota.	In vivo	[213]
Weaning C57Bl/6 mice.	Dietary supplementation with 20% GMP.	Increases the Firmicutes levels; reduces the growth of *Desulfvibrio*, thus increasing the caecal concentrations of SCFAs.	Anti-inflammatory effects.	In vivo	[210]
High-fat diet-fed and streptozotocin-induced type 2 diabetes C57BL/6J mice.	8-week GHP hydrolysate dietary supplementation.	Increases the *Bacteroidetes*/*Firmicutes* ratio; increases the S24-7, *Ruminiclostridium, Blautia*, and *Allobaculum*.	Exerts hypoglycaemic activity; ameliorates dyslipidaemia and inflammation.	In vivo	[215]
Allergen-sensitized rats.	Oral administration of GMP for 17 days.	Increases *Lactobacillus* and *Bifidobacterium* after 3 days of treatment and increases Bacteroides after 17 days of treatment.	Exerts antiallergic activity.	In vivo	[214]
Healthy very preterm infants.	Oral administration of infant formula containing a specific prebiotic mixture 0.65 g scGOS/lcFOS (9:1) and GMP (providing 40 mg sialic acid/100 mL).	Increases *Bifidobacterium*.	Induces health-beneficial microbiota changes resulting in a more efficient production of neuroactive compounds and energy source utilization.	Clinical trial	[217]
Obese postmenopausal women.	Supplementation with 15 g GMP plus 10 g whey protein twice daily for 1 week and thrice daily for 1 week.	Alteration in the faecal microbiota consisting of a reduction of members of the genus *Streptococcus* and of overall α diversity.	Improves satiety and regulates glucose homeostasis.	Clinical trial	[218]
Adult individuals with irritable bowel syndrome.	Three-week period of daily supplementation with 30 g of GMP.	No significant changes in faecal microbiota and faecal immune markers.	No significant effects on inflammation and symptoms of irritable bowel syndrome.	Clinical trial	[221]

## 5. Conclusions

Gut microbiota dysbiosis has been shown to be triggered by various factors, including environmental factors, dietary habits, pharmacological therapies, and diverse health conditions. On the other hand, a dysregulated gut microbiota has been shown to be a crucial factor in the establishment of various health conditions, increasing susceptibility not only to chronic inflammatory bowel diseases but also to several systemic pathologies, including cancer, type II diabetes, and some neurological disorders.

Several studies suggested that a healthy gut microbiota can be restored by bioactive compounds derived from food sources, including milk. The possibility of restoring gut microbiota naturally, by exploiting food resources, is intriguing; however, dietary correction, alone, could take a long time, and a more advantageous possibility would be to use these bioactive food components, individually or in combination with other compounds, as dietary supplements. In this context, several studies have been undertaken to evaluate the effects of single milk bioactive compounds or a combination of them on gut microbiota. Evidence from in vitro and in vivo studies demonstrated the potential of milk oligosaccharides and whey proteins in positively modulating gut microbiota, which have shown to exert a strong prebiotic activity, contributing to restoration of healthy gut microbiota and associated health conditions. Interestingly, these results have been confirmed by clinical trials, performed on both infants and adults suffering from different pathologies and/or undergoing pharmacological therapies. In addition, these studies have helped to elucidate the mechanisms of action linked to the prebiotic activity of these compounds; these include direct prebiotic activity and indirect mechanisms of action mainly related to the selective antimicrobial activity exhibited by some of these bioactive milk components, both of which favour the growth of certain probiotic strains to the detriment of pathogens or other non-beneficial strains.

These findings could open up the possibility of exploiting these molecules as therapeutics for the treatment of gut microbiota disorders and associated pathologies. However, while the results of most of the studies provide plausible evidence for the efficacy of milk oligosaccharides and whey proteins in modulating and restoring a healthy gut microbiota, it must be taken into consideration that many studies have been conducted on animals and that, although studies on humans have given similar results, some of them are divergent. Therefore, further clinical trials are needed to overcome the current limitations and to designate these molecules as effective candidates for the treatment of intestinal dysbiosis in humans.

## Figures and Tables

**Table 3:** In vivo studies and clinical trials on the modulation of gut microbiota by α-lactalbumin.

Patients or Animal Models	Treatment	Effects on Gut Microbiota	Health Effects	Type of Study	Ref.
Potassium Oxonate- and hypoxanthine-induced hyperuricemic mice.	Oral supplementation of α-lactalbumin hydrolysates.	Increases the abundance of some SCFA-producing bacteria; decreases the growth of hyperuricaemia- and inflammation-associated genera.	Reduction in the levels of serum uric acid, creatinine, and urea nitrogen.	In vivo	[177]
Spontaneously hypertensive rats.	Oral gavage of α-lactalbumin hydrolysates under 3 kDa (100 mg/kg BW) and VGINYW (5 mg per kg BW).	Recovers the diversity of the gut microbiota and modulates short-chain fatty acid producing bacteria.	Alleviates the hypertension-associated intestinal microbiota dysbiosis.	In vivo	[178]
High-fat diet-induced NAFLD mice.	Treatment with the α-lactalbumin peptide Asp-Gln-Trp.	Modulates the gut microbiota increasing the ratio of Bacteroides to Firmicutes, reducing the relative abundance of pathogenic bacteria (i.e., *Bacteroides*, *Blautia*, and *Alistipes*) and enhancing the relative abundance of short-chain fatty acid (SCFA)-producing bacteria (i.e., *Muribaculaceae*, *Lachnospiraceae*, and *Rikenellaceae*).	Improves the intestinal barrier integrity and inflammation.	In vivo	[179]
High-fat diet-induced obese mice.	Supplementation of bovine α-lactalbumin hydrolysate.	Modulates the *Bacteroidetes/Firmicutes* ratios and increases the relative abundance of *Lachnospiraceae* and *Blautia*.	Reduces the levels of inflammatory cytokines, such as interleukin-6 and tumour necrosis factor-α, and of lipopolysaccharides. Reduces the obesity-associated systematic inflammation and endotoxaemia.	In vivo	[180]
High-fat diet-fed mice.	Supplementation of dietary α-lactalbumin.	Favours the abundance of the *Lactobacillus*, *Parabacteroides* and *Bifidobacterium* genera.	Decrease in cd36 and glut2 gene expression in the intestine accompanied by increased cumulative energy ingestion.	In vivo	[181]
Preterm pigs (model of newborn infants).	High α-lactalbumin diet.	Improves the growth of *Clostridiaceae, Enterobacteriaceae, Streptococcus*, and *Streptomyces*.	Produces beneficial effects on the growth and functional development of body and organ systems, gut, immunity, and structural brain development.	In vivo	[182]
Full-term infants.	α-Lactalbumin enriched term infant formula plus 3.0 g/L of oligofructose.	Increases the faecal bifidobacteria.	Improves the stool consistency without adversely affecting stool frequency or hydration.	Clinical trial	[183]
Obese and dysmetabolic patients.	Oral supplementation of a combination of inositols, α-lactalbumin, and *Gymnema sylvestre*.	Recovers gut dysbiosis.	Positive impact on insulin, glucose, lipid metabolism.	Clinical trial	[184]

**Table 4:** In vivo studies on the modulation of gut microbiota by lysozyme.

Animal Models	Treatment	Effects on Gut Microbiota	Health Effects	Type of Study	Ref.
Young pigs.	Milk from transgenic goats expressing human-comparable levels of human lysozyme.	Reduction of *coliforms* and *E. coli*.	Reduction in the serum levels of uric acid, creatinine, and urea nitrogen.	In vivo	[196]
Young pigs.	Milk from transgenic goats expressing human-comparable levels of human lysozyme.	Inhibition of pathogenic *E. coli*. Decrease of Firmicutes and increase in Bacteroidetes; reduction of *Clostridia*, *Streptococcaceae*, *Mycobacteriaceae*, and *Campylobacterales*.	Ameliorates intestinal inflammation; accelerates recovery from *E. coli*-induced diarrhoea. Improved gut health.	In vivo	[197,198,199]
Mice with deoxynivalenol-induced depletion of Paneth cells.	Supplementation with lysozyme (200 U/day).	Reduces the Firmicutes/Bacteroidetes ratio; increases the abundance of *Dubosiella* and decreases the abundance of *Lactobacillus*.	Improved Paneth cells’ functionality and gut microbiota restoration.	In vivo	[204]

## Data Availability

Not applicable.
